# Investigation on Recrystallization Channel for Vertical C-Shaped-Channel Nanosheet FETs by Laser Annealing

**DOI:** 10.3390/nano13111786

**Published:** 2023-06-01

**Authors:** Zhuo Chen, Huilong Zhu, Guilei Wang, Qi Wang, Zhongrui Xiao, Yongkui Zhang, Jinbiao Liu, Shunshun Lu, Yong Du, Jiahan Yu, Wenjuan Xiong, Zhenzhen Kong, Anyan Du, Zijin Yan, Yantong Zheng

**Affiliations:** 1 Key Laboratory of Microelectronics Devices & Integrated Technology, Institute of Microelectronics, Chinese Academy of Sciences, Beijing 100029, China; chenzhuo@ime.ac.cn (Z.C.); wangqi@ime.ac.cn (Q.W.); xiaozhongrui@ime.ac.cn (Z.X.); zhangyongkui@ime.ac.cn (Y.Z.); liujinbiao@ime.ac.cn (J.L.); lushunshun@ime.ac.cn (S.L.); duyong@ime.ac.cn (Y.D.); yujiahan@ime.ac.cn (J.Y.); xiongwenjuan@ime.ac.cn (W.X.); kongzhenzhen@ime.ac.cn (Z.K.); duanyan@ime.ac.cn (A.D.); yanzijin@ime.ac.cn (Z.Y.); zhengyantong@ime.ac.cn (Y.Z.); 2 Microelectronics Institute, University of Chinese Academy of Sciences, Beijing 100049, China; 3 Process Integration, Beijing Superstring Academy of Memory Technology, Beijing 100176, China; guilei.wang@bjsamt.org.cn

**Keywords:** vertical channel transistor, self-aligned, laser annealing, recrystallization, Si cap

## Abstract

Transistor scaling has become increasingly difficult in the dynamic random access memory (DRAM). However, vertical devices will be good candidates for 4F^2^ DRAM cell transistors (F = pitch/2). Most vertical devices are facing some technical challenges. For example, the gate length cannot be precisely controlled, and the gate and the source/drain of the device cannot be aligned. Recrystallization-based vertical C-shaped-channel nanosheet field-effect transistors (RC-VCNFETs) were fabricated. The critical process modules of the RC-VCNFETs were developed as well. The RC-VCNFET with a self-aligned gate structure has excellent device performance, and its subthreshold swing (SS) is 62.91 mV/dec. Drain-induced barrier lowering (DIBL) is 6.16 mV/V.

## 1. Introduction

Recently, the technology for the mass production of logic devices has evolved to the 3 nm technology node [1]. In the future, Intel, Samsung, and TSMC will continue to optimize the power, performance, area, and cost (PPAC) for the logic device by using new technologies at the 2 nm technology node, such as gate-all-around FETs (GAAFETs) [2,3] and buried power rail (BPR) [4,5,6,7,8]. However, the scaling of the lateral device has become more and more difficult, and the cost of tape-out has become unaffordable for major design houses. At the same time, vertical devices will be competitive candidates for 4F^2^ cell transistors in the future DRAM device [9,10,11,12,13]. There are many research reports on vertical devices, which can be divided into two routes. The “bottom-up” route enables the growth of vertical nanowire channels by using metal nanoparticle-induced catalysis [14,15]. However, there is a problem with metal elements, such as Au contamination, so it is not compatible with the standard CMOS process. In addition, the “top-down” approach to fabricating vertical transistor devices through lithography and etching processes has been reported by Samsung and IBM [16,17]. However, there are some problems with this route. For example, the device gate length and channel thickness are challenging to control precisely, and the gate cannot be aligned with the source/drain of the vertical device in this route.

In order to solve the above problems, vertical sandwich gate-all-around (GAA) FETs (VSAFETs) based on the SiGe channel are proposed, which have a self-aligned structure between the gate and the source/drain [18,19,20,21]. Recently, vertical C-shaped-channel nanosheet field-effect transistors (VCNFETs) based on the epitaxial process were reported [22,23]. In the process flow of the VCNFETs, the C-shaped SiGe cavity, which is used as a seed layer for the epitaxial growth of the vertical Si channel, is formed through steps of the Si/SiGe/Si superlattice epitaxy and the SiGe selective etching. In this way, the epitaxial process can precisely control the channel thickness of VCNFETs. The device has excellent gate control and current drive performance among similar vertical devices. However, these devices require the expensive Si and SiGe epitaxial processes in the above works. In particular, there are two epitaxial processes in the process flow of the VCNFETs. At the same time, the device introduces Ge elements in the front-end-of-line (FEOL) process, which will increase additional costs in the contamination control of the process equipment.

Therefore, this work proposes a recrystallization-based vertical C-shaped-channel nanosheet field-effect transistors (RC-VCNFETs) and its process integration method. In this method, the substrate Si of the (100) crystal plane is used as a seed layer, and a Si cap layer is grown by a “rapid thermal chemical vapor deposition (RTCVD)” process. The device is subjected to laser annealing so that the amorphous Si cap layer uses the (100) crystal plane of the substrate as a lattice template and recrystallizes vertically upwards to form a high-quality single-crystal Si channel. This method avoids the problem of expensive epitaxial process and contamination of Ge element. Moreover, the RC-VCNFETs have good electrical properties and perfect gate control capability.

## 2. Materials and Methods

**Figure 1:**
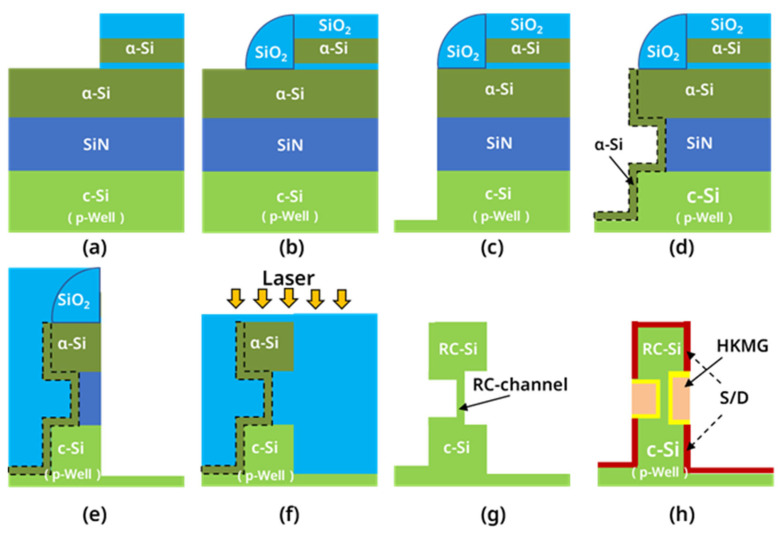
Schematic diagram of the key process steps for the RC-VCNFETs: (**a**) OSO HM formation; (**b**) oxide spacer formation; (**c**) a-Si/SiN/c-Si etching; (**d**) Si cap deposition; (**e**) inner a-Si/SiN/c-Si etching; (**f**) laser annealing; (**g**) STI Recess; (**h**) HKMG etching.

**Figure 2:**
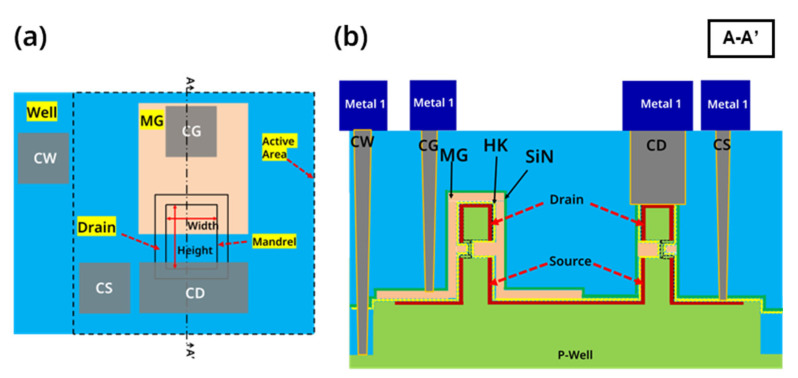
(**a**) Top view and (**b**) cross-sectional view of the device schematic diagram.

**Figure 3:**
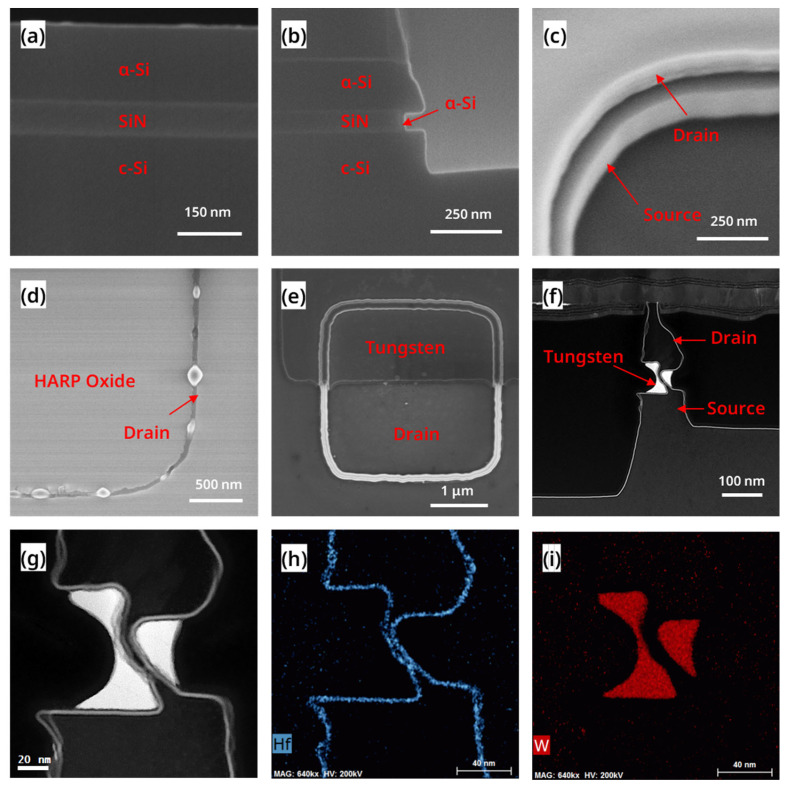
SEM images (**a**) after SiN and a-Si deposition, (**b**) after the growth of Si cap, (**c**) after inner SiN removal, (**d**) after laser annealing (Top view), and (**e**) HKMG etching. (**f**) STEM-HAADF image and (**g**) zoom-in HAADF image of the RC-VCNFET. EDS mapping of (**h**) Hf element and (**i**) W element.

The RC-VCNFETs were prepared on a p-type Si (100) substrate with a resistivity of 8–12 Ohm·cm, and the main process steps are shown in Figure 1a–h. As shown in Figure 2a, there are mandrels with the following sizes: 1 μm × 1 μm, 2 μm × 2 μm, and 4 μm × 4 μm. Firstly, the p-well of the wafer was formed by high-energy boron ion implantation. An 80 nm silicon nitride (SiN) film was deposited on the crystal silicon (c-Si) wafer surface by plasma-enhanced chemical vapor deposition(AMAT Producer S PECVD, Applied Materials, Santa Clara, CA, USA), followed by 180 nm amorphous silicon (a-Si) deposition at 580 °C, as shown in Figure 3a. Then, 10 nm SiO_2_, 300 nm a-Si, and 300 nm SiO_2_ films (abbreviated as OSO stacks) were sequentially deposited by- PECVD. The OSO stack was patterned by I-line photolithography and reactive ion etching (RIE) to form the OSO hard mask (HM) shown in Figure 1a. The top 300 nm SiO_2_ film in the OSO HM was used as the hard mask, the middle 300 nm a-Si film was used as the mandrel, and the bottom 10 nm SiO_2_ was used as the etch stop layer when etching the mandrel by tetramethylammonium hydroxide (TMAH). In the next step, a silicon oxide film was deposited and etched by anisotropy to form silicon oxide sidewalls, as shown in Figure 1b. Then, the OSO HM and oxide spacer were combined into a cap-shaped hard mask.

Furthermore, the a-Si/SiN/c-Si stacks were etched by RIE (TCP9400, Lam Research, Fremont, CA, USA) to form a steep sidewall, as shown in Figure 1c. Afterward, the SiN in the cavity was isotropically etched to form a C-shaped cavity structure, and the c-Si at the bottom of the C-shaped cavity was a seed layer for the next step of the growth of the Si cap by RTCVD (Centura, Applied Materials, Santa Clara, CA, USA). As shown in Figure 1d and Figure 3b, the natural oxide on the surface of the c-Si was removed by the diluted buffered oxide etchant (dBOE). Then, the wafer was immediately loaded into the chamber of the RTCVD equipment, growing a 20 nm Si cap at 580 °C. Meanwhile, the Si cap covering the surface of the OSO cap HM was removed by RIE. Next, the oxide film was deposited with high aspect ratio process. Then, the HARP oxide was polished using chemical mechanical polishing (CMP, FRX200, Ebara, Tokyo, Japan). When HARP was polished to a certain height, the mandrel was exposed. Subsequently, the mandrel was etched by TMAH. Next, 10 nm silicon oxide CESL was etched using RIE. Next, as shown in Figure 1e, the a-Si/SiN/c-Si stacks are etched by RIE. Then, the remaining SiN in the cavity was removed by phosphoric acid solution at 160 °C, as shown in Figure 3c. A 20 nm thick oxide layer was formed on the top of the device with the HARP oxide deposition and the CMP process. As shown in Figure 1f, the wafer surface was irradiated with Nd:YLF pulsed laser (the laser annealing equipment was developed by the Institute of Microelectronics, Chinese Academy of Sciences, the laser’s wavelength is 527 nm, the pulse width is 200 ns, and the frequency is 200 Hz, and the energy density is 1.67 J/cm^2^). At this time, a-Si turned into liquid instantly and then vertically recrystallized to form single crystal silicon channels, and the top view of the device is shown in Figure 3d. During this process, the c-Si inside the SiN cavity served as a lattice template for a-Si recrystallization. As shown in Figure 1g, through the STI Recess step, the oxide on the surface of the device and active area (AA) were removed. The source and drain electrodes of the device were formed by implanting arsenic at a ten-degree angle and phosphorus at a zero-degree angle. Then the high-k metal gate (HKMG) stacks were grown by the atomic layer deposition (ALD, TFS 200, Espoo, Finland) process. After the i-line lithography and etching process, a part of the metal gate was left on AA to serve as a landing pad for the gate contact hole, whose structure is shown in Figure 2a and Figure 3e. At this point, self-aligned metal gates were formed inside a C-shaped cavity on both sides of the recrystallization channel (RC-channel), as shown in Figure 1h and Figure 3f–i. Then CESL SiN film and TEOS were sequentially deposited. Moreover, the interlayer dielectric (ILD) is formed through the CMP process. Subsequently, through the processes of contact hole etching, Ti/TiN liner, tungsten film deposition, and tungsten CMP, four contact plugs, including contact source (CS), contact drain (CD), contact gate (CG), and contact well (CW) are formed. Finally, as shown in Figure 2b, the entire process flow was completed through the processes of PVD deposition, lithography, etching, and alloy annealing to form the Al Pad for interconnection.

Scanning electron microscopy (SEM) was used to observe the topography of the surface and cross-section of the sample, thereby measuring the film thickness and etching depth. Atomic force microscopy (AFM) was used to evaluate the film surface roughness. Transmission electron microscopy (TEM) characterized the device’s component dimensions and crystal structure. Energy-dispersive spectroscopy (EDS) was used to determine the distribution of various elements in the device.

## 3. Results and Discussion

### 3.1. Key Process Module—Si Cap Deposition and Pre-Clean

**Figure 4:**
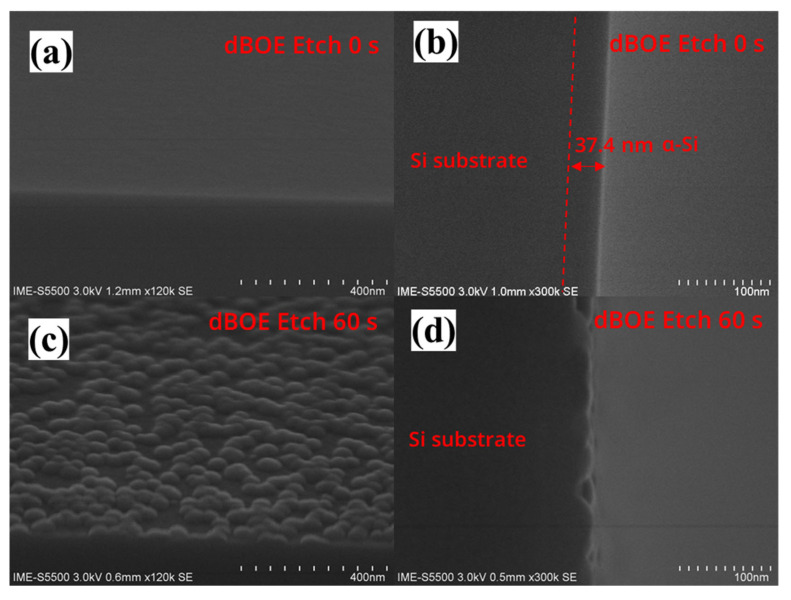
(**a**) Top view SEM image and (**b**) cross-sectional view SEM image of the wafer after the “dBOE cleaning 0 s + Si cap growth” process; (**c**) top view SEM image and (**d**) cross-sectional view SEM image of the wafer after the “dBOE cleaning 60 s + Si cap growth” process.

The RTCVD growth process of the Si cap channel is critical as it relates to subsequent recrystallization processes. Based on the experience of Si and SiGe epitaxial processes, pre-treatment processes are essential for the growth of high-quality crystalline Si channels. Before integrating this process module into the entire process flow, we studied the effect of pre-cleaning on the deposition of the Si cap on a Si (100) substrate. Figure 4a,c show the SEM images of the wafers with p-well after dBOE cleaning for 0 s and 60 s, followed by 40 nm Si cap deposition. Under pre-cleaning conditions for 0 s, the sample surface is very smooth. However, the cross-sectional SEM of Figure 4b shows that the substrate surface has a structure with significantly different contrast, which proves the existence of the a-Si/c-Si interface. Meanwhile, under pre-cleaning conditions for the 60 s, some hillock-like structures are on the sample surface. However, no a-Si/c-Si interface exists in Figure 4d. This result indicates that the crystal structure of the deposited Si cap film is so close to the c-Si at the bottom that the secondary electron signal cannot distinguish them.

**Figure 5:**
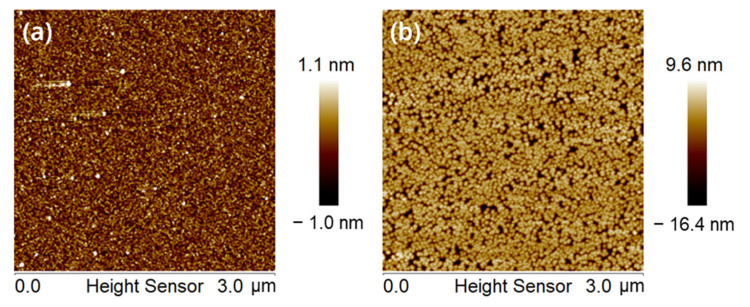
AFM images of the surface of (**a**) the wafer after the “dBOE cleaning 0 s + Si cap growth” process and (**b**) the wafer after the “dBOE cleaning 60 s + Si cap growth” process.

Figure 5a,b show the AFM images of wafers after 0 s and 60 s of dBOE cleaning, followed by 40 nm Si cap deposition. The RMS of the sample surface pre-cleaned for 60 s is 1.89 nm, and the maximum height of the surface protrusions is 14.9 nm, which shows that although the 60 s dBOE removes the natural oxide layer on the surface of Si (100) wafer. There may still be some defects on the surface of the wafer that cannot be removed by dBOE etching. These defects result in the existence of these hillock-like structures on the surface of the wafer. Some processes, such as well implantation, may introduce these defects. In order to further optimize the quality of the film and the yield of the device in the future, it may be necessary to further clarify the root causes of these defects.

**Figure 6:**
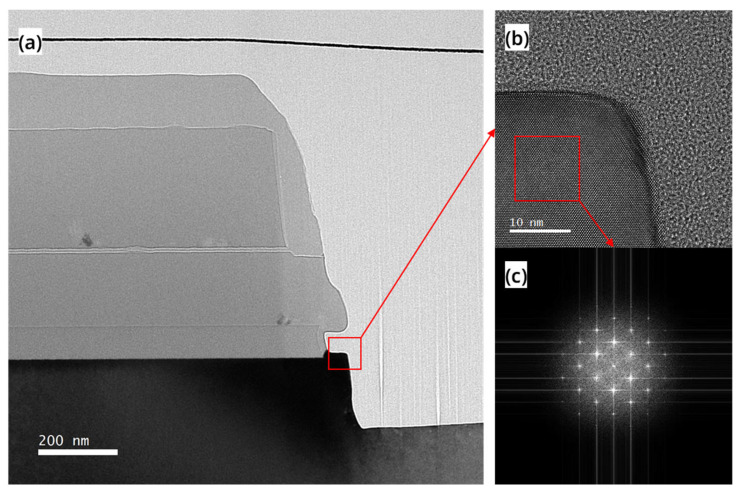
(**a**) TEM image, (**b**) HRTEM image, and (**c**) FFT image of the cross-section of the device after the Si cap growth.

In addition, we also analyzed the structure of the final devices. Figure 6a shows a cross-sectional view TEM image of the RC-VCNFETs, and its structure is shown in Figure 1d. It can be found that the Si cap film deposited by RTCVD has excellent conformality and can well fill the SiN cavity with a width of 80 nm. Figure 6b is a high-resolution transmission electron microscope (HRTEM) image of the sample. A clear Si lattice shows that the Si film that grows on the c-Si seed layer in the SiN cavity is very close to the single crystal structure. As shown in Figure 6c, the Fourier transform is performed on the red box area in Figure 6b, and the diffraction pattern of the silicon (110)-oriented planes is obtained, which is the same as the crystal plane index in the wafer notch direction.

### 3.2. Structure of RC-Channel

**Figure 7:**
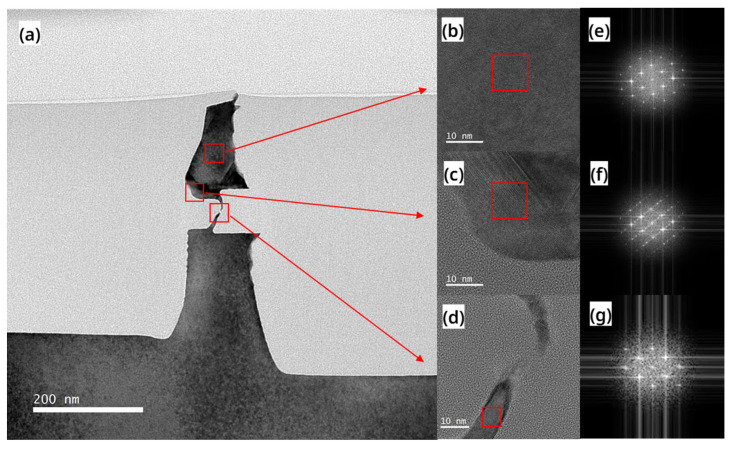
(**a**) TEM image, (**b**–**d**) HRTEM images, and (**e**–**g**) FFT images of the cross-section of the device after laser annealing process.

To further evaluate the crystal quality of the RC-channel, we conducted a TEM analysis on the devices after laser annealing. Figure 7a shows that most areas of the RC-VCNFET device contrast similarly with the c-Si substrate in the TEM image, indicating that the RC-channel is high-quality recrystallized Si. HRTEM images in Figure 7b,d also confirm the presence of high-quality recrystallized Si, while Fourier transform results in Figure 7e,g show that this area is a (110) crystal plane. However, as shown in Figure 7a, darker regions at the top of the RC-VCNFET device suggest some defects. As shown in Figure 7c, there is a dislocation along the Si (111) direction at the lower left corner area of the top Si. The Fourier transform result in Figure 7f also indicates twinning defects exist in this area. In addition, it should be emphasized that there are fine cracks in the HARP oxide on the top of the device in Figure 7a, which indicates that the a-Si tends to expand upwards during the process of transitioning from liquid to solid under the pulsed laser, which is consistent with the SEM image in Figure 3d. In Figure 1f, the HARP oxide encapsulating RC-VCNFET device may negatively affect the recrystallization process of the a-Si. Therefore, adjusting HARP oxide height may improve the crystal quality of the Si cap layer in the RC-VCNFETs.

### 3.3. Characterization and Materials Analysis of the Device

**Figure 8:**
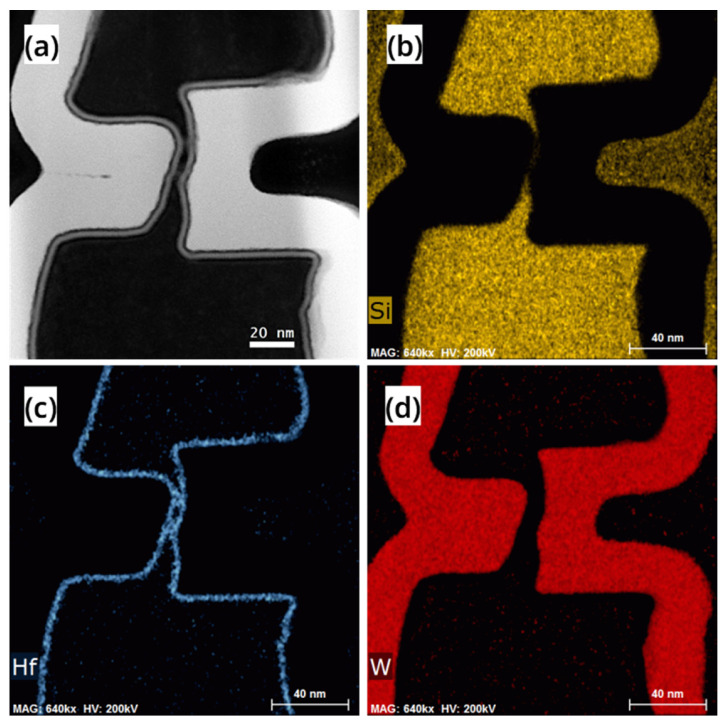
(**a**) HAADF image, (**b**) Si element, (**c**) Hf element, (**d**) W element mapping of the cross-section of the device.

After completing all the process steps, FIB-TEM analysis and electrical performance testing were performed on a RC-VCNFET. Figure 8 shows the high-angle annular dark-field (HAADF) image and EDS mapping of the channel region of the device with HKMG. The Si in black contrast in Figure 8a shows the integrity of the RC-VCNFET device’s channel, and the channel’s minimum thickness was measured to be 3.8 nm. Figure 8b indicates that the thickness of the RC-channel is less than the limited EDS spatial resolution. That means the channel is very thin, so that the device will have a strong gate control capability. Figure 8c,d show that HKMG deposited by ALD has an excellent conformal filling ability for tens-of-nanometer-scale C-shaped cavities. Moreover, the gate length of the RC-VCNFET is about 49 nm, which is defined by the thickness of the tungsten filled in the gap on the left side of the RC-VCNFET.

**Figure 9:**
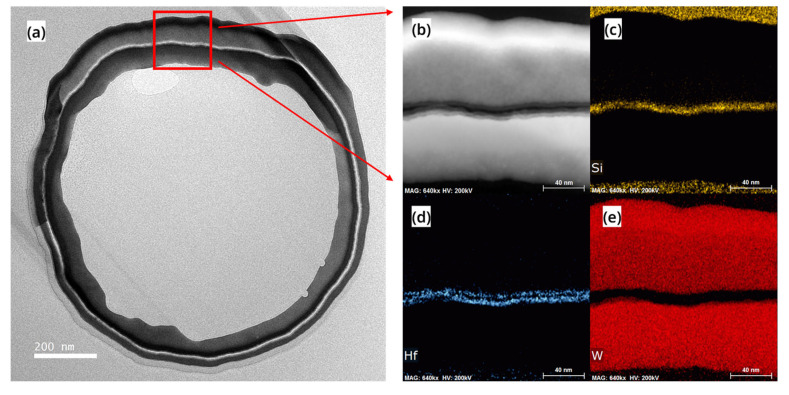
(**a**) TEM image, (**b**) HAADF image, (**c**) Si element, (**d**) Hf element, (**e**) W element mapping of the top view of the device.

Figure 9a shows the top view TEM image of the device at the channel height, and the design size of the mandrel layer is 1 μm × 1 μm. The white contrast Si in Figure 9a shows the continuity of the device channel in the plane direction. Due to the diffraction effect of light, the 1 μm × 1 μm rectangular mandrel is significantly distorted at the corners, making the square mandrel on the original layout an approximately circular structure. The actual channel width of this RC-VCNFET is measured to be about 2.76 μm, and its original design value on the layout is 4 μm. Figure 9b,c also show the continuity of the RC-VCNFET device channel with an average thickness of about 7.74 nm. Figure 9d,e also prove that the ALD-grown HfO_2_ and tungsten films have perfect conformality at the nanoscale.

### 3.4. Electrical Properties of the Device

**Figure 10:**
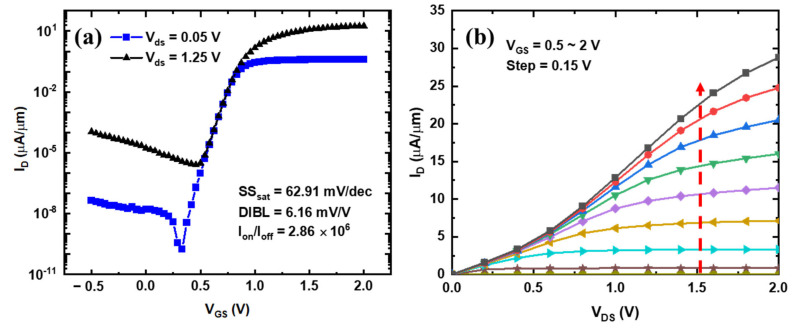
(**a**) Transfer characteristic curve and (**b**) output characteristic curve of the device.

Figure 10a,b show the Id-Vg and Id-Vd curves of the RC-VCNFET device, respectively, where I_on_ is 15.7 μA/μm (I_D_ @ V_OV_ = V_G_ − V_T_ = 1 V, V_DS_ = 1.25 V), I_off_ is 5.48 × 10^−6^ pA/μm (I_D_ @ V_OV_ = V_G_ − V_T_ =−0.50 V, V_DS_ = 1.25 V), threshold voltage V_T_ is 0.75 V, subthreshold swing (SS) is 62.91 mV/dec, drain-induced barrier lowering (DIBL) is 6.16 mV/V, and the on–off ratio is 2.86 × 10^6^. The above electrical parameters show that the current driving performance of the current device is relatively low. However, the device’s off-state leakage current and gate control capability are excellent, which can be attributed to the fact that the thickness of the RC-channel is only 3.8 nm. It should be noted that these electrical characteristics only show the driving performance of the RC-VCNFET fabricated in the early stage. The device’s performance can be further improved by optimizing the laser annealing recrystallization process, source/drain contact, and other process modules.

## 4. Conclusions

The RC-VCNFETs with a self-aligned structure were proposed and fabricated in this work. Its related process modules were developed: selective etching of SiN cavity, channel Si cap growth, and recrystallization channel formation. The RC-VCNFET was characterized by TEM, EDS, and other analysis methods. Finally, the electrical characteristics of the device were tested and analyzed. The device showed superior gate control capability, with a SS of 62.91 mV/dec and a DIBL of 6.16 mV/V.

## Data Availability

The data presented in this study are available on request from the corresponding author.
